# Autophagy as a possible mechanism for micronutrient remobilization from leaves to seeds

**DOI:** 10.3389/fpls.2014.00011

**Published:** 2014-01-24

**Authors:** Mathieu Pottier, Céline Masclaux-Daubresse, Kohki Yoshimoto, Sébastien Thomine

**Affiliations:** ^1^Institut des Sciences du Végétal-UPR2355, Saclay Plant Sciences, CNRS, Gif-sur-YvetteFrance; ^2^Institut Jean-Pierre Bourgin-UMR1318, Saclay Plant Sciences, Institut National de la Recherche AgronomiqueVersailles, France; ^3^Institut Jean-Pierre Bourgin-UMR1318, Saclay Plant Sciences, AgroParisTech, VersaillesFrance

**Keywords:** transition metal, isotopic labeling, nutrient use efficiency, leaf senescence, nutrient fluxes, *atg*, Fe, Zn

## Abstract

Seed formation is an important step of plant development which depends on nutrient allocation. Uptake from soil is an obvious source of nutrients which mainly occurs during vegetative stage. Because seed filling and leaf senescence are synchronized, subsequent mobilization of nutrients from vegetative organs also play an essential role in nutrient use efficiency, providing source-sink relationships. However, nutrient accumulation during the formation of seeds may be limited by their availability in source tissues. While several mechanisms contributing to make leaf macronutrients available were already described, little is known regarding micronutrients such as metals. Autophagy, which is involved in nutrient recycling, was already shown to play a critical role in nitrogen remobilization to seeds during leaf senescence. Because it is a non-specific mechanism, it could also control remobilization of metals. This article reviews actors and processes involved in metal remobilization with emphasis on autophagy and methodology to study metal fluxes inside the plant. A better understanding of metal remobilization is needed to improve metal use efficiency in the context of biofortification.

## INTRODUCTION

Micronutrients, such as metals, are essentials for cell functions. Zinc (Zn), which exists only as divalent cation, plays an important role in protein structure and function thank to its Lewis acids properties. Transition metals such as iron (Fe), copper (Cu), or manganese (Mn), which have unpaired electrons that promote their involvement in oxido-reduction reactions, are used in a wealth of biological processes (Pierre and Fontecave, 1999). A third of the proteins characterized at the structural level are metalloproteins, highlighting the need of metals for cell functions (Finney and O’Halloran, 2003).

In plants, transition metal functions are mainly associated to energy production mechanisms, thereby about 80% of Fe in mesophyll cell is localized in chloroplasts (Nouet et al., 2011). Fe is essential for chlorophyll synthesis, nitrogen fixation, DNA replication, reactive oxygen species (ROS) detoxification, and electron transport chain in both mitochondria and chloroplasts (Nouet et al., 2011; Yruela, 2013). Mn plays a central role in the photosystem II (PS II) where it catalyzes water oxidation (Tommos et al., 1998). This element is also involved in sugar metabolism, Mn-superoxide dismutase (SOD), and chloroplastic enzymes such as decarboxylases and dehydrogenases (Luk and Culotta, 2001; Horsburgh et al., 2002; Aggarwal et al., 2012). Cu is integrated into plastocyanines involved in electron transfer of chloroplasts (Yruela, 2013). It plays also an essential role in the cytochrome oxidase of mitochondria (Bleackley and Macgillivray, 2011). Zn is required for carbon fixation through the carbonic anhydrase (Badger and Price, 1994). It is also needed for the Cu/Zn-SOD, transcriptional regulation by zinc-finger DNA binding proteins and for the turnover of PSII in chloroplasts (Kurepa et al., 1997; Bleackley and Macgillivray, 2011; Lu et al., 2011). Therefore, plants need metals to achieve vital functions in all their organs.

Among all plant organs, seed is a special one because it has to store metals required for germination and during the first days of seedling development. Hence in annual plants, seed formation is a crucial step in which plant sacrifices itself to store nutrients for its offspring. Seed filling depends on nutrient originating from *de novo* uptake by roots or remobilization from senescent organs.

Here, we review genes and processes involved in metal remobilization during seed filling. We will discuss methodologies that can be used to study metal fluxes in plants and thereby determine the relative contribution of uptake and remobilization pathways. Autophagy is a ubiquitous process involved in cellular nutrient recycling. Because it was recently shown to play a critical role in nitrogen remobilization (Htwe et al., 2011; Guiboileau et al., 2012), this review focuses on autophagy as a potential mechanism to make metal available for subsequent remobilization during senescence.

## ORIGIN OF SEED METALS: UPTAKE FROM SOIL VS REMOBILIZATION FROM SENESCENT TISSUES

### CIRCULATION OF METALS INTO THE PLANT AND MICRONUTRIENT USE EFFICIENCY

Understanding metal seed filling requires knowledge on the general micronutrient pathways which was already summarized in several recent reviews (Pittman, 2005; Palmgren et al., 2008; Morrissey and Guerinot, 2009; Puig and Peñarrubia, 2009; Yruela, 2009; Pilon, 2011; Waters and Sankaran, 2011; Thomine and Vert, 2013).

**FIGURE 1:**
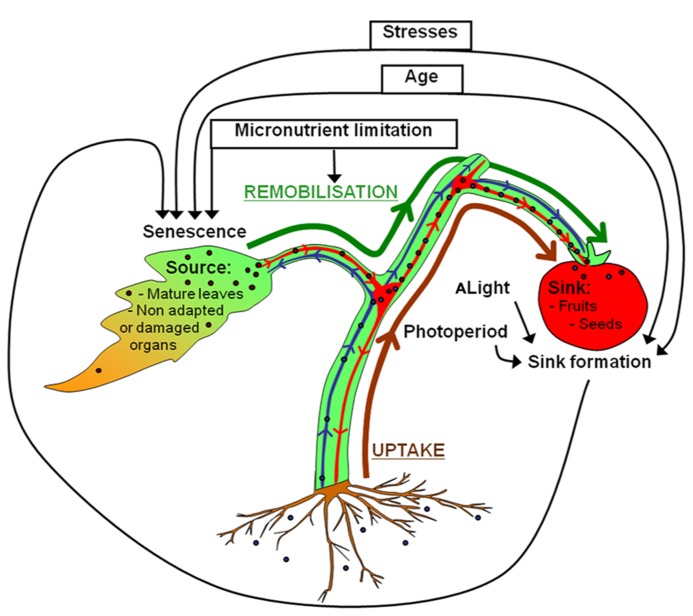
**Uptake and remobilization pathways involved in seed filling with emphasis on source-sink relationships.** Micronutrients from the rhizosphere (brown arrow) are taken up into roots and transported to the xylem vessels (shown in blue). After xylem loading, micronutrients are translocated into shoots for subsequent unloading. Micronutrients located in the xylem can also be unloaded into the xylem parenchyma of nodes to be transferred to phloem vessels (shown in red) by specific transporters (Sondergaard et al., 2004; Tanaka et al., 2008; Yamaji and Ma, 2009). This is essential for seed filling which is only achieved by the phloem sap (Patrick and Offler, 2001). Phloem micronutrients are unloaded to fill seeds. Because seed filling is also achieved by nutrient remobilized from senescent tissues (green arrow), seed formation requires close synchronization between sink formation and source organ senescence. Age, biotic and abiotic stresses contribute to orchestrate nutrient mobilization during leaf senescence with the formation of reproductive organs and seed filling (black arrows). Light and photoperiod act indirectly on leaf senescence by stimulating the development of the reproductive organs.

On the whole, both uptake from soil and remobilization from senescent organs may participate in metal loading in seeds (**Figure 1**). To date, little is known about the contribution of metal remobilization from senescent organs to seed filling. In contrast, this topic is well documented regarding nitrogen. It was shown that uptake and fixation of nitrogen dramatically decrease at the onset of reproductive stage in cereals, oilseed rape and legumes (Salon et al., 2011). Accordingly, 50 to 90% of nitrogen grain of rice, wheat, or maize originate from leaf remobilization (Masclaux et al., 2001). This highlights that the importance of nitrogen remobilization for seed filling is conserved in most plants. However, some species, such as oilseed rape, have a low nitrogen remobilization capacity resulting in low nitrogen use efficiency (Schjoerring et al., 1995; Etienne et al., 2007).

As for nitrogen, it is necessary to better understand metal remobilization from senescent organs during seed filling with the aim to increase micronutrient use efficiency in the context of intensive agriculture, fertilization limitations, and biofortification. This is especially important as metal availability may become limiting under certain environmental conditions (drought, low temperature) and soil characteristics (low metal content, high salt content, ionic unbalance, low pH, high bicarbonate concentration; Chen and Barak, 1982; Karamanos et al., 1986; Graham, 1988; Alloway, 2009).

### METHODOLOGIES TO DETERMINE NUTRIENT FLUX

The most common way to study nutrient fluxes within the plant is to determine the “apparent remobilization” which consists in the measurement of the total amount of element of interest present in different plant organs at different times (Masclaux-Daubresse et al., 2010). However, this approach does not provide sufficient resolution and does not allow distinguishing nutrients coming from different pathways, such as nutrient uptake from soil and nutrient remobilization from senescent leaves.

The most appropriate approach to study short-term accumulation, uptake from soil and fluxes between tissues is the use of isotopes as tracers. Isotopic labeling can be implemented with different protocols (Grusak, 1994; Wu et al., 2010; Erenoglu et al., 2011; Hegelund et al., 2012).

Metal fluxes may be monitored by pulse-chase labeling using radioactive or stable isotopes. The ^59^Fe, ^65^Zn, and ^68^Zn radioisotopes have been used for pulse labeling on specific organs followed by a chase period to facilitate the identification of source organs contributing to seed filling in peas, wheat and rice (Grusak, 1994; Wu et al., 2010; Erenoglu et al., 2011; Zheng et al., 2012). Following this approach, it was demonstrated that nutrient supply can affect Zn remobilization in wheat (Erenoglu et al., 2011). In rice, differences in Zn remobilization efficiency between genotypes were observed using isotopic pulse-chase on specific organs (Wu et al., 2010).

Recently, pulse labeling using very short life ß^+^ radioisotope like ^52^Fe, ^52^Mn, and ^62^Zn has been used to image metal fluxes within a plant *via* a real-time and non-destructive technique called Positron-Emitting Tracer Imaging System (Kume et al., 1997; Tsukamoto et al., 2006; Tsukamoto et al., 2009).

Non-radioactive isotope is also used for pulse labeling on specific organs. Application of ^65^Cu to one individual leaf of rice allowed to study Cu redistribution between the different leaves during vegetative stage (Zheng et al., 2012). Non-radioactive isotopes can be also added in the nutrient solution for labeling plants early during development in order to monitor nutrient movement during vegetative stages or later at reproductive stage to study remobilization and seed filling. Using Zn isotopes, this pulse-chase approach has been used to quantify the effect of nutrient limitation on Zn fluxes between organs in rice and wheat (Wu et al., 2010; Erenoglu et al., 2011). Moreover, ^70^Zn pulse-chase labeling combined with laser ablation-inductively coupled plasma-mass spectrometry has provided a spatial distribution of Zn within wheat seeds revealing zinc transport barriers during grain filling in wheat (Wang et al., 2010).

Long term labeling in nutrient solution may be performed to address the contribution of uptake from soil to organs during a specific developmental stage, with respect to the contribution of endogenous remobilization. Continuous application of ^68^Zn provided evidence that Zn uptake before anthesis contributes to more than 50% to the total Zn grain content in rice (Wu et al., 2010). Shorter continuous labeling can also be used to determine the uptake capacity by measuring isotope accumulation in roots (Hegelund et al., 2012) or isotope depletion in the nutritive solution (Erenoglu et al., 2011).

Isotopic labeling is an essential tool to study metal fluxes within the plant but require the availability of enriched isotopes and adequate analytical tools. Initially, isotopic labeling was mainly performed using radioactive isotope despite the risk for humans. Nowadays, enriched stable isotopes are more and more accessible at least for Fe, Ni, Cu, Zn, and Mo. They represent a healthier and less restrictive alternative but their analysis requires the use of mass spectrometry, such as inductively coupled plasma-mass spectrometry.

## THE COUPLING BETWEEN SENESCENCE AND MICRONUTRIENT REMOBILIZATION

### CONTROL OF SENESCENCE AND REMOBILIZATION AT THE WHOLE PLANT LEVEL

**FIGURE 2:**
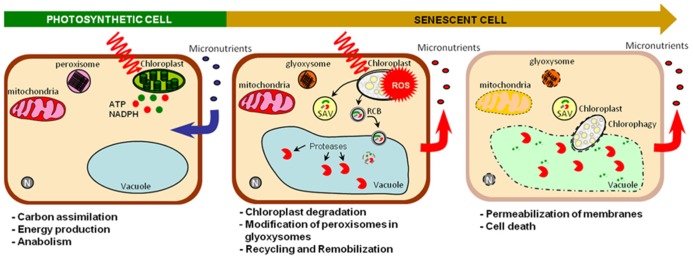
**Sink/source transition at the cellular level.** Active photosynthetic cells perform carbon fixation, energy production and anabolism and require micronutrients for these functions. Senescence modifies these sink cells into a source cells undergoing catabolism. Intense catabolism activities and nutrient recycling occurs in chloroplasts, cytosol, and vacuole allowing nutrient remobilization. Chloroplasts, which concentrate a large part of metals, are first affected (Zavaleta-Mancera et al., 1999). Pigment degradation directly takes place in chloroplasts (Hörtensteiner et al., 1995; Park et al., 2007). However, stromal proteins are degradated into the central vacuole through rubisco containing body (RCB: autophagosome) or into senescence associated vacuoles (SAV) through an ATG-independent route which is not well understand yet (Hörtensteiner and Feller, 2002; Ishida et al., 2008; Ishida et al., 2013). These dismantling mechanisms decrease chloroplast seize enabling whole chloroplast degradation via chlorophagy (Ishida et al., 2013). Peroxisomes are modified to glyoxysomes, which produce energy and soluble sugars from lipid catabolism (Buchanan-Wollaston, 1997; del Rìo et al., 1998). Mitochondria that remain intact until late after senescence onset, are in turn degraded when the energy demand decreases (Yoshida, 2003). Finally, membrane permeabilization causes loss of cytoplasm that finally leads to death. ROS, reactive oxygen species; SAV, senescence-associated vacuoles; RCB, rubisco containing body; N, nucleus.

Senescence is an active process controlled by age whereby sink tissues performing photosynthesis and anabolism become source tissues undergoing catabolism (**Figure 2**). Senescence makes nutrients available for further plant organs (Hörtensteiner and Feller, 2002), contributing to nutrient use efficiency. Optimal remobilization requires close synchronization between sink formation and source organ senescence (**Figure 1**). It was observed that the removal of sink tissues delays senescence in oilseed rape, soybean and wheat and decrease nitrogen remobilization in oilseed rape and soybean (Patterson and Brun, 1980; Crafts-Brandner and Egli, 1987; Noquet et al., 2004; Htwe et al., 2011). However, senescence and remobilization are also controlled by other parameters such as nutrient availability (**Figure 1**). In *Arabidopsis*, nitrogen limitation triggers leaf senescence (Lemaître et al., 2008). In wheat, remobilization of Fe and Zn from flag leaves to seeds is increased under nutrient-limiting conditions (Waters et al., 2009; Wu et al., 2010; Sperotto et al., 2012b). Conversely continuous nutrient uptake during seed formation may account for low nutrient remobilization in some species (Masclaux-Daubresse and Chardon, 2011; Waters and Sankaran, 2011). However, an opposite behavior was observed in barley plants for which remobilization increased upon high Zn supply. This illustrates the diversity of Zn management at the whole plant level (Hegelund et al., 2012). Moreover, other abiotic and biotic stresses such as pathogen attack, high salinity, drought, low temperature, modifications of light intensity, and quality can also cause premature senescence and remobilization (Nooden et al., 1996; Buchanan-Wollaston, 1997; Gan and Amasino, 1997). Because they are sessile, plants developed high plasticity to respond to environment conditions, triggering cell death and remobilization in order to save nutrients and produce more adapted organs and tissues.

### CONTROL OF SENESCENCE AND REMOBILIZATION AT THE MOLECULAR LEVEL

Transcript analysis, comparing green and senescing leaves, led to the identification of senescence-associated genes (SAG) in different species (Hensel et al., 1993; Buchanan-Wollaston, 1994; Smart et al., 1995; Guo et al., 2004; Buchanan-Wollaston et al., 2005; van der Graaff et al., 2006; Breeze et al., 2011). Irrevocably, the expression of genes encoding cysteine proteases is strongly induced in senescent leaves (Hensel et al., 1993; Smart et al., 1995; Bhalerao et al., 2003; Andersson et al., 2004; Guo et al., 2004; Breeze et al., 2011). As expected, these analyses confirmed induction of genes involved in hormonal pathways (Andersson et al., 2004; van der Graaff et al., 2006; Breeze et al., 2011). Indeed, senescence is regulated by the balance between senescence promoting hormones, namely jasmonic acid, abscisic acid, salicylic acid, and ethylene, and senescence repressing hormones such as cytokinins, auxins, and gibberellins (van der Graaff et al., 2006). As hormones, sugars are known to act as signaling molecules and several lines of evidence indicate that they also contribute to senescence regulation. Sugar concentrations rise in senescent leaves. Moreover, overexpression of hexokinase, a sugar sensor, accelerates senescence whereas anti-sense expression delays senescence in *Arabidopsis* (Nooden et al., 1997; Masclaux et al., 2000; Xiao et al., 2000; Watanabe et al., 2013).

Genes coding metal ion binding proteins such as metallothioneins, ferritins, zinc-finger proteins, metalloproteases (Ftsh) and metal transporters were also frequently found to be upregulated in senescent leaves (Buchanan-Wollaston, 1994; Bhalerao et al., 2003; Andersson et al., 2004; Guo et al., 2004; Zelisko et al., 2005). This may illustrate the involvement of metals in degradation mechanisms and/or the importance of their remobilization (Breeze et al., 2011). Furthermore, these transcriptomic analyses highlighted the significant induction of autophagy related genes (*ATG* genes) and genes encoding NAC and WRKY transcription factors (Andersson et al., 2004; Guo et al., 2004; van der Graaff et al., 2006; Breeze et al., 2011). Whereas NAC have already been demonstrated to be involved in micronutrient remobilization during senescence (Olmos et al., 2003; Guo and Gan, 2006; Uauy et al., 2006; Sperotto et al., 2009, 2010; Waters et al., 2009), nothing is known about the implication of *ATG* genes in this process.

## ROLE OF AUTOPHAGY IN NUTRIENT RECYCLING AND REMOBILIZATION

### INVOLVEMENT OF AUTOPHAGY IN NUTRIENT RECYCLING

Autophagy catabolizes cytoplasmic components that are no longer useful. It eliminates aberrant proteins and damaged organelles for the maintenance of essential cellular function by vacuole internalization mediated by double membrane vesicles called autophagosomes (Yoshimoto, 2012). Genes involved in autophagy (*ATG*) were first defined by a genetic screen in yeast (Matsuura et al., 1997), thereby molecular mechanisms have been well described on this organism (for reviews see Thompson and Vierstra, 2005; Bassham, 2007; Li and Vierstra, 2012; Yoshimoto, 2012). Most of these genes turned out to have conserved functions in all eukaryotic cells. They encode proteins involved in the induction of autophagy, membrane delivery for autophagosome formation, nucleation, expansion, and enclosure of autophagosomes (Thompson and Vierstra, 2005).

Autophagy can be triggered upon nutrient starvation and stress leading to intracellular remodeling, which allows plants to respond to environmental constraints (Yoshimoto, 2012). Accordingly, mutants impaired in *ATG* genes exhibit decreased growth associated with premature senescence when they develop under carbon or nitrogen starvation (Doelling et al., 2002; Hanaoka et al., 2002; Yoshimoto et al., 2004; Phillips et al., 2008; Chung et al., 2010; Suttangkakul et al., 2011). Plants defective in autophagy are thus unable to cope with nutrient starvation suggesting that autophagy is an important mechanism for nutrient use efficiency and cellular homeostasis.

### AUTOPHAGY CONTROLS NUTRIENT REMOBILIZATION DURING SENESCENCE

During senescence, cytoplasmic components such as organelles are gradually dismantled and degraded. Autophagy is an essential degradation process for nutrient recycling and remobilization. Accordingly, up-regulation of *ATG* genes is observed during leaf senescence in *Arabidopsis* (Doelling et al., 2002; van der Graaff et al., 2006; Chung et al., 2010; Breeze et al., 2011) and the decrease of chloroplast number and chloroplast size during senescence is affected in *Arabidopsis* *atg4a4b-1* mutant (Wada et al., 2009).

Because of its key role in the degradation of cellular components during nutrient recycling and its up-regulation and involvement during senescence, it was hypothesized that autophagy could play a role in nutrient remobilization. During senescence, autophagy was shown to be involved in the degradation of chloroplasts and specifically of RuBisCO which is the most abundant leaf protein containing about 80% of the cellular nitrogen (**Figure 2**; Chiba et al., 2003; Ishida et al., 2008; Wada et al., 2009; Guiboileau et al., 2012; Ishida et al., 2013). In addition, pulse-chase experiments in which ^15^N labeling was applied in nutrient solution during vegetative stage revealed a significant decrease of nitrogen remobilization from vegetative tissues to seeds in *atg* mutants. These results demonstrated that autophagy is required for nitrogen remobilization and seed filling (Guiboileau et al., 2012).

Chloroplast is the organelle where metals are most intensively used. Thereby about 80% of the cellular Fe is localized in chloroplasts (Nouet et al., 2011). Because autophagy is involved in the degradation of organelles, including chloroplasts, the role of autophagy in metal recycling in source tissues for remobilization to the seeds has to be considered. In plants, autophagy leads to the degradation of autophagosome cargo within the vacuole. Hence, tonoplastic metal efflux transporters are needed to retrieve metals from the vacuole. Interestingly, transcriptomic analyses that highlight autophagy induction during senescence in *Arabidopsis* leaf also show specific up-regulation of *NRAMP3*, a gene encoding a transporter involved in metal mobilization from vacuoles (Thomine et al., 2003; Lanquar et al., 2005, 2010; Breeze et al., 2011). Availability of metals in source tissues may therefore also be dependent on autophagy and subsequent mobilization from vacuole during senescence.

## REMOBILIZATION AND AUTOPHAGY IN THE CONTEXT OF BIOFORTIFICATION

### BIOFORTIFICATION TO IMPROVE HUMAN DIET

Key micronutrients are often not sufficiently available in human diet (Kennedy et al., 2003). Over 60% of the world population are Fe deficient and over 30% are Zn deficient (White and Broadley, 2009). Staple food crops such as cereal grains are poor sources of some mineral nutrients, including Fe and Zn. Thus, the importance of cereals in human diet accounts in large part for micronutrients deficiencies (Gomez-Galera et al., 2010).

Biofortification aims at increasing the availability of key micronutrients such as Fe and Zn in crops (White and Broadley, 2009). For this purpose, conventional breeding and genetic engineering are performed in rice, which is the major staple crop in most countries affected by Fe-deficiency (Juliano, 1993; WHO, 2002; Sperotto et al., 2012a). Single or multiple metal homeostasis genes were already introduced in rice through genetic engineering to improve grain Fe content (Sperotto et al., 2012a). By pyramiding transgenes conferring strong sink strength in seeds, high metal translocation and enhancing phloem unloading during seed maturation, it was possible to increase Fe concentration by 4.4 in rice seeds (Masuda et al., 2012).

### ENGINEERING AUTOPHAGY AS A NEW WAY FOR BIOFORTIFICATION

Another option to increase seed micronutrient content could be to improve their availability in source tissues for remobilization during seed formation. Himelblau and Amasino (2001) showed that senescence of *Arabidopsis* leaves only leads to a decrease by 40% of leaf concentrations of metals such as Mo, Fe, Cu, and Zn. Thus, about 60% of these micronutrients are not remobilized and can therefore not participate to seed filling. Up-regulating autophagy in source tissues specifically during seed formation could improve intracellular nutrient recycling and thereby increase the nutrient pool available for reallocation. However, because autophagy is not specific, this approach may increase seed yield without increasing Zn or Fe concentrations. To improve seed quality, up-regulation of autophagy should be combined with a strategy that specifically targets a metal, such as the expression of ferritin under the control of a seed endosperm promoter in the case of Fe (Sperotto et al., 2012a).

More than thirty genes are involved in autophagy (Yoshimoto, 2012). It might therefore not be straightforward to increase autophagy by overexpressing autophagy related genes during seed formation. However, autophagy is regulated at the post-transcriptional level by the target of rapamycin (TOR) kinase complex (Noda and Ohsumi, 1998; Kamada et al., 2000). Because TOR is a negative regulator of autophagy, its specific inhibition in vegetative tissues during seed formation may be the best approach to stimulate autophagy and nutrient recycling. On the other hand, TOR kinase complex is not a specific regulator of autophagy. It controls many others aspect of metabolism (Diaz-Troya et al., 2008). Besides, autophagy itself is not only involved in nutrient recycling. It also controls the hypersensitive response (Yoshimoto et al., 2009). Therefore, further investigations are necessary to determine if TOR inactivation during senescence is efficient for biofortification and to identify more specific regulators.

## Conflict of Interest Statement

The authors declare that the research was conducted in the absence of any commercial or financial relationships that could be construed as a potential conflict of interest.

## References

[B1] Aggarwal A. Sharma I. Tripathi B. N. Munjal A. K. Baunthiyal M. Sharma V. (2012). “Metal toxicity and photosynthesis,” in *Photosynthesis: Overviews on Recent Progress and Future Perspectives* edsItoh S. Mohanty P. Guruprasad K. N. (New Delhi:IK International Publishing House (Pvt) Limited).

[B2] Alloway B. J. (2009). Soil factors associated with zinc deficiency in crops and humans. *Environ. Geochem. Health* 31 537–548 10.1007/s10653-009-9255-4 19291414

[B3] Andersson A. Keskitalo J. Sjodin A. Bhalerao R. Sterky F. Wissel K. (2004). A transcriptional timetable of autumn senescence. *Genome Biol.* 5 R24 10.1186/gb-2004-5-4-r24 PMC395783 15059257

[B4] Badger M. R. Price G. D. (1994). The role of carbonic anhydrase in photosynthesis. *Annu. Rev. Plant Biol.* 45 369–392 10.1146/annurev.pp.45.060194.002101

[B5] Bassham D. C. (2007). Plant autophagy-more than a starvation response. *Curr. Opin. Plant Biol.* 10 587–593 10.1016/j.pbi.2007.06.006 17702643

[B6] Bhalerao R. Keskitalo J. Sterky F. Erlandsson R. Björkbacka H. Birve S. J. (2003). Gene expression in autumn leaves. *Plant Physiol.* 131 430–442 10.1104/pp.012732 12586868 PMC166820

[B7] Bleackley M. R Macgillivray R. T. A. (2011). Transition metal homeostasis: from yeast to human disease. *Biometals* 24 785–809 10.1007/s10534-011-9451-4 21479832

[B8] Breeze E. Harrison E. Mchattie S. Hughes L. Hickman R. Hill C. (2011). High-resolution temporal profiling of transcripts during *Arabidopsis* leaf senescence reveals a distinct chronology of processes and regulation. *Plant Cell* 23 873–894 10.1105/tpc.111.083345 21447789 PMC3082270

[B9] Buchanan-Wollaston V. (1994). Isolation of cDNA clones for genes that are expressed during leaf senescence in *Brassica napus* (identification of a gene encoding a senescence-specific metallothionein-like protein). *Plant Physiol.* 105 839–846 10.1104/pp.105.3.839 8058836 PMC160730

[B10] Buchanan-Wollaston V. (1997). The molecular biology of leaf senescence. *J. Exp. Bot.* 48 181–199 10.1093/jxb/48.2.181

[B11] Buchanan-Wollaston V. Page T. Harrison E. Breeze E. Lim P. O. Nam H. G. (2005). Comparative transcriptome analysis reveals significant differences in gene expression and signalling pathways between developmental and dark/starvation-induced senescence in *Arabidopsis*. *Plant J.* 42 567–585 10.1111/j.1365-313X.2005.02399.x 15860015

[B12] Chen Y. Barak P. (1982). Iron nutrition of plants in calcareous soils. *Adv. Agron.* 35 217–240 10.1016/S0065-2113(08)60326-0

[B13] Chiba A. Ishida H. Nishizawa N. K. Makino A. Mae T. (2003). Exclusion of ribulose-1, 5-bisphosphate carboxylase/oxygenase from chloroplasts by specific bodies in naturally senescing leaves of wheat. *Plant Cell Physiol.* 44 914–921 10.1093/pcp/pcg118 14519773

[B14] Chung T. Phillips A. R. Vierstra R. D. (2010). ATG8 lipidation and ATG8-mediated autophagy in *Arabidopsis* require ATG12 expressed from the differentially controlled ATG12A AND ATG12B loci. *Plant J.* 62 483–493 10.1111/j.1365-313X.2010.04166.x 20136727

[B15] Crafts-Brandner S. J. Egli D. B. (1987). Sink removal and leaf senescence in soybean: cultivar effects. *Plant Physiol.* 85 662–666 10.1104/pp.85.3.662 16665756 PMC1054318

[B16] del Rìo L. A. Pastori G. M. Palma J. M. Sandalio L. M. Sevilla F. Corpas F. J. (1998). The activated oxygen role of peroxisomes in senescence. *Plant Physiol.* 116 1195–1200 10.1104/pp.116.4.1195 9536035 PMC1539175

[B17] Diaz-Troya S. Pérez-Pérez M. E. Florencio F. J. Crespo J. L. (2008). The role of TOR in autophagy regulation from yeast to plants and mammals. *Autophagy* 4 851–865 18670193 10.4161/auto.6555

[B18] Doelling J. H. Walker J. M. Friedman E. M. Thompson A. R. Vierstra R. D. (2002). The APG8/12-activating enzyme APG7 is required for proper nutrient recycling and senescence in *Arabidopsis thaliana*. *J. Biol. Chem.* 277 33105–33114 10.1074/jbc.M204630200 12070171

[B19] Erenoglu E. B. Kutman U. B. Ceylan Y. Yildiz B. Cakmak I. (2011). Improved nitrogen nutrition enhances root uptake, root-to-shoot translocation and remobilization of zinc (65Zn) in wheat. *New Phytol.* 189 438–448 10.1111/j.1469-8137.2010.03488.x 21029104

[B20] Etienne P. Desclos M. Le Gou L. Gombert J. Bonnefoy J. Maurel K. (2007). N-protein mobilisation associated with the leaf senescence process in oilseed rape is concomitant with the disappearance of trypsin inhibitor activity. *Funct. Plant Biol.* 34 895–906 10.1071/FP07088 32689418

[B21] Finney L. A O’Halloran T. V. (2003). Transition metal speciation in the cell: insights from the chemistry of metal ion receptors. *Sci. Signal.* 300 931 10.1126/science.1085049 12738850

[B22] Gan S. Amasino R. M. (1997). Making sense of senescence (molecular genetic regulation and manipulation of leaf senescence). *Plant Physiol.* 113 313 10.1104/pp.113.2.313 PMC158144 12223609

[B23] Gomez-Galera S. Rojas E. Sudhakar D. Zhu C. Pelacho A. M. Capell T. (2010). Critical evaluation of strategies for mineral fortification of staple food crops. *Transgenic Res.* 19 165–180 10.1007/s11248-009-019311-y 19685153

[B24] Graham R D. (1988). “Genotypic diferences in tolerance to manganese deficiency,” in *Manganese in Soils and Plants* eds Graham R. D. Hannam R. J. Uren N. C. (Dordrecht: Kluwer Academic Publishers) 261–276

[B25] Grusak M. A. (1994). Iron transport to developing ovules of *Pisum sativum* (I. Seed import characteristics and phloem iron-loading capacity of source regions). *Plant Physiol.* 104 649–655 10.1104/pp.104.2.649 12232115 PMC159243

[B26] Guiboileau A. Yoshimoto K. Soulay F. Bataillé M.-P. Avice J.-C. (2012). Autophagy machinery controls nitrogen remobilization at the whole-plant level under both limiting and ample nitrate conditions in *Arabidopsis*. *New Phytol.* 194 732–740 10.1111/j.1469-8137.2012.04084.x 22404536

[B27] Guo Y. Cai Z. Gan S. (2004). Transcriptome of *Arabidopsis* leaf senescence. *Plant Cell Environ.* 27 521–549 10.1111/j.1365-3040.2003.01158.x

[B28] Guo Y. Gan S. (2006). AtNAP, a NAC family transcription factor, has an important role in leaf senescence. *Plant J.* 46 601–612 10.1111/j.1365-313X.2006.02723.x 16640597

[B29] Hanaoka H. Noda T. Shirano Y. Kato T. Hayashi H. Shibata D. (2002). Leaf senescence and starvation-induced chlorosis are accelerated by the disruption of an *Arabidopsis* autophagy gene. *Plant Physiol.* 129 1181–1193 10.1104/pp.011024 12114572 PMC166512

[B30] Hegelund J. N. Pedas P. Husted S. Schiller M. Schjoerring J. K. (2012). Zinc fluxes into developing barley grains: use of stable Zn isotopes to separate root uptake from remobilization in plants with contrasting Zn status. *Plant Soil* 361 241–250 10.1007/s11104-012-1272-x

[B31] Hensel L. L. Grbic V. Baumgarten D. A. Bleecker A. B. (1993). Developmental and age-related processes that influence the longevity and senescence of photosynthetic tissues in *Arabidopsis*. *Plant Cell* 5 553–564 10.1105/tpc.5.5.553 8518555 PMC160293

[B32] Himelblau E. Amasino R. M. (2001). Nutrients mobilized from leaves of *Arabidopsis thaliana* during leaf senescence. *J. Plant Physiol.* 158 1317–1323 10.1078/0176-1617-00608

[B33] Horsburgh M. J. Wharton S. J. Karavolos M. Foster S. J. (2002). Manganese: elemental defence for a life with oxygen. *Trends Microbiol.* 10 496–501 10.1016/S0966-842X(02)02462-9 12419613

[B34] Hörtensteiner S. Feller U. (2002). Nitrogen metabolism and remobilization during senescence. *J. Exp. Bot.* 53 927–937 10.1093/jexbot/53.370.927 11912235

[B35] Hörtensteiner S. Vicentini F. Matile P. (1995). Chlorophyll breakdown in senescent cotyledons of rape, *Brassica napus* L: enzymatic cleavage of pheophorbide a in vitro. *New Phytol.* 129 237–246 10.1111/j.1469-8137.1995.tb04293.x 33874553

[B36] Htwe N. M. P. S., Yuasa T. Ishibashi Y. Tanigawa H. Okuda M. Zheng S.-H. (2011). Leaf senescence of soybean at reproductive stage is associated with induction of autophagy-related genes, GmATG8c, GmATG8i and GmATG4. *Plant Prod. Sci.* 14 141–147 10.1626/pps.14.141

[B37] Ishida H. Izumi M. Wada S. Makino A. (2013). Roles of autophagy in chloroplast recycling. *Biochim. Biophys. Acta* 10.1016/j.bbabio.2013.11.009 [Epub ahead of print] 24269172

[B38] Ishida H. Yoshimoto K. Izumi M. Reisen D. Yano Y. Makino A. (2008). Mobilization of rubisco and stroma-localized fluorescent proteins of chloroplasts to the vacuole by an ATG gene-dependent autophagic process. *Plant Physiol.* 148 142–155 10.1104/pp.108.122770 18614709 PMC2528122

[B39] Juliano B. O. (1993). *Rice in Human Nutrition*. Rome: International Rice Research Institute & FAO

[B40] Kamada Y. Funakoshi T. Shintani T. Nagano K. Ohsumi M. Ohsumi Y. (2000). Tor-mediated induction of autophagy via an Apg1 protein kinase complex. *J. Cell Biol.* 150 1507–1513 10.1083/jcb.150.6.1507 10995454 PMC2150712

[B41] Karamanos R. E. Kruger G. A Stewart J. W. B. (1986). Copper deficiency in cereal and oilseed crops in northern Canadian prairie soils. *Agron. J.* 78 317–323 10.2134/agronj1986.00021962007800020021x

[B42] Kennedy G. Nantel G. Shetty P. (2003). The scourge of “hidden hunger”: global dimensions of micronutrient deficiencies. *Food Nutr. Agric.* 8–16.

[B43] Kume T. Matsuhashi S. Shimazu M. Ito H. Fujimura T. Adachi K. (1997). Uptake and transport of positron-emitting tracer (18 F) in plants. *Appl. Radiat. Isot.* 48 1035–1043 10.1016/S0969-8043(97)00117-6

[B44] Kurepa J. Hérouart D. Van Montagu M Inzé D. (1997). Differential expression of CuZn-and Fe-superoxide dismutase genes of tobacco during development, oxidative stress, and hormonal treatments. *Plant Cell Physiol.* 38 463–470 10.1093/oxfordjournals.pcp.a029190 9177032

[B45] Lanquar V. Lelievre F. Bolte S. Hames C. Alcon C. Neumann D. (2005). Mobilization of vacuolar iron by AtNRAMP3 and AtNRAMP4 is essential for seed germination on low iron. *EMBO J.* 24 4041–4051 10.1038/sj.emboj.7600864 16270029 PMC1356305

[B46] Lanquar V. Ramos M. S. Lelièvre F. Barbier-Brygoo H. Krieger-Liszkay A. Kramer U. (2010). Export of vacuolar manganese by AtNRAMP3 and AtNRAMP4 is required for optimal photosynthesis and growth under manganese deficiency. *Plant Physiol.* 152 1986–1999 10.1104/pp.109.150946 20181755 PMC2850043

[B47] Lemaître T. Gaufichon L. Boutet-Mercey S. Christ A. Masclaux-Daubresse C. (2008). Enzymatic and metabolic diagnostic of nitrogen deficiency in *Arabidopsis thaliana* Wassileskija accession. *Plant Cell Physiol.* 49 1056–1065 10.1093/pcp/pcn081 18508804

[B48] Li F. Vierstra R. D. (2012). Autophagy: a multifaceted intracellular system for bulk and selective recycling. *Trends Plant Sci.* 17 526–537 10.1016/j.tplants.2012.05.006 22694835

[B49] Lu Y. Hall D. A. Last R. L. (2011). A small zinc finger thylakoid protein plays a role in maintenance of photosystem II in *Arabidopsis thaliana*. *Plant Cell* 23 1861–1875 10.1105/tpc.111.085456 21586683 PMC3123961

[B50] Luk E. E. C. Culotta V. C. (2001). Manganese superoxide dismutase in *Saccharomyces cerevisiae* acquires its metal co-factor through a pathway involving the Nramp metal transporter, Smf2p. *J. Biol. Chem.* 276 47556–47562 10.1074/jbc.M108923200 11602606

[B51] Masclaux-Daubresse C. Daniel-Vedele F. Dechorgnat J. Chardon F. Gaufichon L. Suzuki A. (2010). Nitrogen uptake, assimilation and remobilization in plants: challenges for sustainable and productive agriculture. *Ann. Bot.* 105 1141–1157 10.1093/aob/mcq028 20299346 PMC2887065

[B52] Masclaux-Daubresse C. L. Chardon F. (2011). Exploring nitrogen remobilization for seed filling using natural variation in *Arabidopsis thaliana*. *J. Exp. Bot.* 62 2131–2142 10.1093/jxb/erq405 21273332 PMC3060690

[B53] Masclaux C. Quilleré I. Gallais A. Hirel B. (2001). The challenge of remobilisation in plant nitrogen economy. A survey of physio-agronomic and molecular approaches. *Ann. Appl. Biol.* 138 69–81 10.1111/j.1744-7348.2001.tb00086.x

[B54] Masclaux C. Valadier M.-H. Brugière N. Morot-Gaudry J.-F. Hirel B. (2000). Characterization of the sink/source transition in tobacco (*Nicotiana tabacum* L.) shoots in relation to nitrogen management and leaf senescence. *Planta* 211 510–518 10.1007/s004250000310 11030550

[B55] Masuda H. Ishimaru Y. Aung M. S. Kobayashi T. Kakei Y. Takahashi M. (2012). Iron biofortification in rice by the introduction of multiple genes involved in iron nutrition. *Sci. Rep.* 2 543 10.1038/srep00543 PMC3408131 22848789

[B56] Matsuura A. Tsukada M. Wada Y. Ohsumi Y. (1997). Apg1p, a novel protein kinase required for the autophagic process in *Saccharomyces cerevisiae*. *Gene* 192 245–250 10.1016/S0378-1119(97)00084-X 9224897

[B57] Morrissey J. Guerinot M. L. (2009). Iron uptake and transport in plants: the good, the bad, and the ionome. *Chem. Rev.* 109 4553–4567 10.1021/cr900112r 19754138 PMC2764373

[B58] Noda T. Ohsumi Y. (1998). Tor, a phosphatidylinositol kinase homologue, controls autophagy in yeast. *J. Biol. Chem.* 273 3963–3966 10.1074/jbc.273.7.3963 9461583

[B59] Nooden L. D. Guiamet J. J. John I. (1997). Senescence mechanisms. *Physiol. Plant.* 101 746–753 10.1111/j.1399-3054.1997.tb01059.x

[B60] Nooden L. D. Hillsberg J. W. Schneider M. J. (1996). Induction of leaf senescence in *Arabidopsis thaliana* by long days through a light-dosage effect. *Physiol. Plant.* 96 491–495 10.1111/j.1399-3054.1996.tb00463.x

[B61] Noquet C. Avice J.-C. Rossato L. Beauclair P. Henry M.-P. Ourry A. (2004). Effects of altered source-sink relationships on N allocation and vegetative storage protein accumulation in *Brassica napus* L. *Plant Sci.* 166 1007–1018 10.1016/j.plantsci.2003.12.014

[B62] Nouet C. Motte P. Hanikenne M. (2011). Chloroplastic and mitochondrial metal homeostasis. *Trends Plant Sci.* 16 395–404 10.1016/j.tplants.2011.03.005 21489854

[B63] Olmos S. Distelfeld A. Chicaiza O. Schlatter A. R. Fahima T. Echenique V. (2003). Precise mapping of a locus affecting grain protein content in durum wheat. *Theor. Appl. Genet.* 107 1243–1251 10.1007/s00122-003-1377-y 12923624

[B64] Palmgren M. G. Clemens S. Williams L. E. Kramer U. Borg S. Schjorring J. K. (2008). Zinc biofortification of cereals: problems and solutions. *Trends Plant Sci.* 13 464–473 10.1016/j.tplants.2008.06.005 18701340

[B65] Park S.-Y. Yu J.-W. Park J.-S. Li J. Yoo S.-C. Lee N.-Y. (2007). The senescence-induced staygreen protein regulates chlorophyll degradation. *Plant Cell* 19 1649–1664 10.1105/tpc.106.044891 17513504 PMC1913741

[B66] Patrick J. W. Offler C. E. (2001). Compartmentation of transport and transfer events in developing seeds. *J. Exp. Bot.* 52 551–564 10.1093/jexbot/52.356.551 11373304

[B67] Patterson T. G. Brun W. A. (1980). Influence of sink removal in the senescence pattern of wheat. *Crop Sci.* 20 19–23 10.2135/cropsci1980.0011183X002000010006x

[B68] Phillips A. R. Suttangkakul A. Vierstra R. D. (2008). The ATG12-conjugating enzyme ATG10 is essential for autophagic vesicle formation in *Arabidopsis thaliana*. *Genetics* 178 1339–1353 10.1534/genetics.107.086199 18245858 PMC2278079

[B69] Pierre J. L. Fontecave M. (1999). Iron and activated oxygen species in biology: the basic chemistry. *Biometals* 12 195–199 10.1023/A:1009252919854 10581682

[B70] Pilon M. (2011). Moving copper in plants. *New Phytol.* 192 305–307 10.1111/j.1469-8137.2011.03869.x 21950332

[B71] Pittman J. K. (2005). Managing the manganese: molecular mechanisms of manganese transport and homeostasis. *New Phytol.* 167 733–742 10.1111/j.1469-8137.2005.01453.x 16101910

[B72] Puig S Peñarrubia L. (2009). Placing metal micronutrients in context: transport and distribution in plants. *Curr. Opin. Plant Biol* 12 299–306 10.1016/j.pbi.2009.04.008 19481498

[B73] Salon C. Avice J. C. Alain O. Prudent M. Voisin A.-S. (2011). “Plant N fluxes and modulation by nitrogen, heat, and water stresses: a review based on comparison of legumes and non legume plants,” in *Abiotic Stress in Plants – Mechanisms and Adaptations* eds Shanker A. Venkateswarlu B. (Rijeka: InTech) 78–118

[B74] Schjoerring J. K. Bock J. G. H. Gammelvind L. Jensen C. R. Mogensen V. O. (1995). Nitrogen incorporation and remobilization in different shoot components of field-grown winter oilseed rape (*Brassica napus* L.) as affected by rate of nitrogen application and irrigation. *Plant Soil* 177 255–264 10.1007/BF00010132

[B75] Smart C. M. Hosken S. E. Thomas H. Greaves J. A. Blair B. G. Schuch W. (1995). The timing of maize leaf senescence and characterisation of senescence-related cDNAs. *Physiol. Plant.* 93 673–682 10.1111/j.1399-3054.1995.tb05116.x

[B76] Sondergaard T. E. Schulz A. Palmgren M. G. (2004). Energization of transport processes in plants. Roles of the plasma membrane H+-ATPase. *Plant Physiol.* 136 2475–2482 10.1104/pp.104.048231 PMC523315 15375204

[B77] Sperotto R. A. Boff T. Duarte G. L. Santos L. S. Grusak M. A. Fett J. P. (2010). Identification of putative target genes to manipulate Fe and Zn concentrations in rice grains. *J. Plant Physiol.* 167 1500–1506 10.1016/j.jplph.2010.05.003 20580124

[B78] Sperotto R. A. Ricachenevsky F. K. Duarte G. L. Boff T. Lopes K. L. Sperb E. R. (2009). Identification of up-regulated genes in flag leaves during rice grain filling and characterization of OsNAC5, a new ABA-dependent transcription factor. *Planta* 230 985–1002 10.1007/s00425-009-1000-9 19697058

[B79] Sperotto R. A. Ricachenevsky F. K. Waldow V. D. A. Fett J. P. (2012a). Iron biofortification in rice: it’s a long way to the top. *Plant Sci.* 190 24–39 10.1016/j.plantsci.2012.03.004 22608517

[B80] Sperotto R. A. Vasconcelos M. W. Grusak M. A. Fett J. P. (2012b). Effects of different Fe supplies on mineral partitioning and remobilization during the reproductive development of rice (*Oryza sativa* L.). *Rice* 5 1–11 10.1186/1939-8433-5-27 PMC4883723 24279875

[B81] Suttangkakul A. Li F. Chung T. Vierstra R. D. (2011). The ATG1/ATG13 protein kinase complex is both a regulator and a target of autophagic recycling in *Arabidopsis*. *Plant Cell* 23 3761–3779 10.1105/tpc.111.090993 21984698 PMC3229148

[B82] Tanaka M. Wallace I. S. Takano J. Roberts D. M. Fujiwara T. (2008). NIP6; 1 is a boric acid channel for preferential transport of boron to growing shoot tissues in *Arabidopsis*. *Plant Cell* 20 2860–2875 10.1105/tpc.108.058628 18952773 PMC2590723

[B83] Thomine S. Lelièvre F. Debarbieux E. Schroeder J. I. Barbier-Brygoo H. (2003). AtNRAMP3, a multispecific vacuolar metal transporter involved in plant responses to iron deficiency. *Plant J.* 34 685–695 10.1046/j.1365-313X.2003.01760.x 12787249

[B84] Thomine S. Vert G. (2013). Iron transport in plants: better be safe than sorry. *Curr. Opin. Plant Biol* 16 322–327 10.1016/j.pbi.2013.01.003 23415557

[B85] Thompson A. R. Vierstra R. D. (2005). Autophagic recycling: lessons from yeast help define the process in plants. *Curr. Opin. Plant Biol* 8 165–173 10.1016/j.pbi.2005.01.013 15752997

[B86] Tommos C. Hoganson C. W. Di Valentin M. Lydakis-Simantiris N. Dorlet P. Westphal K. (1998). Manganese and tyrosyl radical function in photosynthetic oxygen evolution. *Curr. Opin. Chem. Biol* 2 244–252 10.1016/S1367-5931(98)80066-5 9667938

[B87] Tsukamoto T. Nakanishi H. Kiyomiya S. Watanabe S. Matsuhashi S. Nishizawa N. K. (2006). 52Mn translocation in barley monitored using a positron-emitting tracer imaging system. *Soil Sci. Plant Nutr.* 52 717–725 10.1111/j.1747-0765.2006.00096.x

[B88] Tsukamoto T. Nakanishi H. Uchida H. Watanabe S. Matsuhashi S. Mori S. (2009). 52Fe Translocation in barley as monitored by a positron-emitting tracer imaging system (PETIS): evidence for the direct translocation of Fe from roots to young leaves via phloem. *Plant Cell Physiol.* 50 48–57 10.1093/pcp/pcn192 19073647 PMC2638711

[B89] Uauy C. Distelfeld A. Fahima T. Blechl A. Dubcovsky J. (2006). A NAC gene regulating senescence improves grain protein, zinc, and iron content in wheat. *Science* 314 1298–1301 10.1126/science.1133649 17124321 PMC4737439

[B90] van der Graaff E. Schwacke R. Schneider A. Desimone M. Flugge U.-I. Kunze R. (2006). Transcription analysis of *Arabidopsis* membrane transporters and hormone pathways during developmental and induced leaf senescence. *Plant Physiol.* 141 776–792 10.1104/pp.106.079293 16603661 PMC1475451

[B91] Wada S. Ishida H. Izumi M. Yoshimoto K. Ohsumi Y. Mae T. (2009). Autophagy plays a role in chloroplast degradation during senescence in individually darkened leaves. *Plant Physiol.* 149 885–893 10.1104/pp.108.130013 19074627 PMC2633819

[B92] Wang Y. X. Specht A. Horst W. J. (2010). Stable isotope labelling and zinc distribution in grains studied by laser ablation ICP-MS in an ear culture system reveals zinc transport barriers during grain filling in wheat. *New Phytol.* 189 428–437 10.1111/j.1469-8137.2010.03489.x 20946419

[B93] Watanabe M. Balazadeh S. Tohge T. Erban A. Giavalisco P. Kopka J. (2013). Comprehensive dissection of spatio-temporal metabolic shifts in primary, secondary and lipid metabolism during developmental senescence in *Arabidopsis thaliana*. *Plant Physiol.* 162 1290–1310 10.1104/pp.113.217380 23696093 PMC3707545

[B94] Waters B. M. Sankaran R. P. (2011). Moving micronutrients from the soil to the seeds: genes and physiological processes from a biofortification perspective. *Plant Sci.* 180 562–574 10.1016/j.plantsci.2010.12.003 21421405

[B95] Waters B. M. Uauy C. Dubcovsky J. Grusak M. A. (2009). Wheat (*Triticum aestivum*) NAM proteins regulate the translocation of iron, zinc, and nitrogen compounds from vegetative tissues to grain. *J. Exp. Bot.* 60 4263–4274 10.1093/jxb/erp257 19858116

[B96] White P. J. Broadley M. R. (2009). Biofortification of crops with seven mineral elements often lacking in human diets – iron, zinc, copper, calcium, magnesium, selenium and iodine. *New Phytol.* 182 49–84 10.1111/j.1469-8137.2008.02738.x 19192191

[B97] WHO. (2002). *World Health Report 2002: World Health Report: Reducing Risks to Health Noncommunicable Diseases*. Geneva: World Health Organization

[B98] Wu C.-Y. Lu L.-L. Yang X.-E. Feng Y. Wei Y.-Y. Hao H.-L. (2010). Uptake, translocation, and remobilization of zinc absorbed at different growth stages by rice genotypes of different Zn densities. *J. Agric. Food Chem.* 58 6767–6773 10.1021/jf100017e 20481473

[B99] Xiao W. Sheen J. Jang J.-C. (2000). The role of hexokinase in plant sugar signal transduction and growth and development. *Plant Mol. Biol.* 44 451–461 10.1023/A:1026501430422 11197321

[B100] Yamaji N. Ma J. F. (2009). A transporter at the node responsible for intervascular transfer of silicon in rice. *Plant Cell* 21 2878–2883 10.1105/tpc.109.069831 19734433 PMC2768918

[B101] Yoshida S. (2003). Molecular regulation of leaf senescence. *Curr. Opin. Plant Biol* 6 79–84 10.1016/S1369526602000092 12495755

[B102] Yoshimoto K. (2012). Beginning to understand autophagy, an intracellular self-degradation system in plants. *Plant Cell Physiol.* 53 1355–1365 10.1093/pcp/pcs099 22764279

[B103] Yoshimoto K. Hanaoka H. Sato S. Kato T. Tabata S. Noda T. (2004). Processing of ATG8s, ubiquitin-like proteins, and their deconjugation by ATG4s are essential for plant autophagy. *Plant Cell* 16 2967–2983 10.1105/tpc.104.025395 15494556 PMC527192

[B104] Yoshimoto K. Jikumaru Y. Kamiya Y. Kusano M. Consonni C. Panstruga R. (2009). Autophagy negatively regulates cell death by controlling NPR1-dependent salicylic acid signaling during senescence and the innate immune response in *Arabidopsis*. *Plant Cell* 21 2914–2927 10.1105/tpc.109.068635 19773385 PMC2768913

[B105] Yruela I. (2009). Copper in plants: acquisition, transport and interactions. *Funct. Plant Biol.* 36 409–430 10.1071/FP08288 32688656

[B106] Yruela I. (2013). Transition metals in plant photosynthesis. *Metallomics* 5 1090–1109 10.1039/C3MT00086A 23739807

[B107] Zavaleta-Mancera H. Franklin K. Ougham H. Thomas H. Scott I. (1999). Regreening of senescent *Nicotiana* leaves. II. Redifferenciation of plastids. *J. Exp. Biol.* 50 1683–1689 10.1093/jxb/50.340.1683

[B108] Zelisko A. Garcia-Lorenzo M. Jackowski G. Jansson S. Funk C. (2005). AtFtsH6 is involved in the degradation of the light-harvesting complex II during high-light acclimation and senescence. *Proc. Natl. Acad. Sci. U.S.A.* 102 13699–13704 10.1073/pnas.0503472102 16157880 PMC1224624

[B109] Zheng L. Yamaji N. Yokosho K. Ma J. F. (2012). YSL16 is a phloem-localized transporter of the copper-nicotianamine complex that is responsible for copper distribution in rice. *Plant Cell* 24 3767–3782 10.1105/tpc.112.103820 23012434 PMC3480301

